# Supplementary material for: JMJD1B-mediated FEN1 demethylation allows timely switching of Okazaki fragment maturation core enzymes to avoid mutagenic flap ligation by PARP1-LIG3
Source: bioRxiv. 2025 Oct 6:2025.10.06.680735. Preprint. [Version 1] doi: 10.1101/2025.10.06.680735 (PMC12632346; doi:10.1101/2025.10.06.680735)
Supplement: Supplement 1 [file NIHPP2025.10.06.680735v1-supplement-1.pdf]

Supplemental information bioRxiv preprint doi: https://doi.org/10.1101/2025.10.06.680735; this version posted October 6, 2025. The copyright holder for this preprint (which was not certified by peer review) is the author/funder, who has granted bioRxiv a license to display the preprint in perpetuity. It is made available under aCC-BY 4.0 International license.

Supplementary Table S1: List of proteins in the FEN1 complexes identified by LC-MS/MS analysis

Rank	Protein
1	FEN1 Flap endonuclease 1
2	PCNA Proliferating cell nuclear antigen
3	DDX46 DEAD (Asp-Glu-Ala-Asp) box polypeptide 46 (DDX46)
4	PRKDC Isoform 1 of DNA-dependent protein kinase catalytic subunit
5	POLDIP3 Polymerase delta interacting protein 46
6	UBC;RPS27A;UBB ubiquitin and ribosomal protein S27a precursor
7	CMBL Carboxymethylenebutenolidase homolog
8	CCT2 T-complex protein 1 subunit beta
9	RPN1 Dolichyl-diphosphooligosaccharide--protein glycosyltransferase 67 kDa subunit precursor
10	VIM Vimentin
11	NAIF1 Isoform 1 of Nuclear apoptosis-inducing factor 1
12	RNA-directed DNA polymerase (Reverse transcriptase), related domain containing protein
13	SLC1A5 Neutral amino acid transporter B(0)
14	INPP4B Type II inositol-3,4-bisphosphate 4-phosphatase
15	PRPSAP2 Phosphoribosyl pyrophosphate synthetase-associated protein 2
16	SIPA1L2 Isoform 1 of Signal-induced proliferation-associated 1-like protein 2
17	RBM38 14 kDa protein
18	ARSK Arylsulfatase K
19	FAM131A Isoform 2 of Protein FAM131A
20	MFN1 Isoform 2 of Mitofusin-1
21	ADAM28 Isoform 1 of ADAM 28
22	KCTD2 KCTD2 protein (Fragment)
23	DCD Dermcidin
24	ANXA5 Annexin A5
25	ATP1A2 Sodium/potassium-transporting ATPase subunit alpha-2
26	MAD1L1 Mitotic spindle assembly checkpoint protein MAD1
27	Uncharacterized protein ENSP00000357890 (Fragment)
28	CKAP5 Isoform 1 of Cytoskeleton-associated protein 5
29	BCAP31 B-cell receptor-associated protein 31
30	TBR1 T-box brain protein 1
31	SEPT1 Septin-1
32	SRBD1 Isoform 1 of S1 RNA-binding domain-containing protein 1
33	UACA Uveal autoantigen with coiled-coil domains and ankyrin repeats
34	MFSD9 Major facilitator superfamily domain-containing protein 9
35	ASH1L Probable histone-lysine N-methyltransferase ASH1L
36	ZMYM5 Isoform 1 of Zinc finger MYM-type protein 5
37	JMJD1B Isoform 1 of JmjC domain-containing histone demethylation protein 3B
38	FRG1 Protein FRG1
39	GCN1L1 Translational activator GCN1
40	EPPK1 epiplakin 1
41	NOL6 Isoform 1 of Nucleolar protein 6
42	MEGF8 Isoform 1 of Multiple epidermal growth factor-like domains 8
43	GNAL guanine nucleotide binding protein (G protein), alpha activating activity polypeptide, 1
44	LRP1 Prolow-density lipoprotein receptor-related protein 1
45	FGD1 FYVE, RhoGEF and PH domain-containing protein 1
46	PTPRT Isoform 1 of Receptor-type tyrosine-protein phosphatase T
47	CLTCL1 Isoform 1 of Clathrin heavy chain 2
48	MED8 Isoform 2 of Mediator of RNA polymerase II transcription subunit 8
49	SAMD4A SAMD4A protein
50	PRO2049
51	NBL1 Similar to Collagen alpha-1(VIII) chain precursor
52	GP1BA platelet glycoprotein Ib alpha polypeptide precursor
53	CERK Ceramide kinase
54	RAB5B Ras-related protein Rab-5B
55	OBSCN Isoform 1 of Obscurin
56	PDIA6 Isoform 2 of Protein disulfide-isomerase A6
57	DYNC1H1 Cytoplasmic dynein 1 heavy chain 1
58	VPS36 Isoform 1 of Vacuolar protein-sorting-associated protein 36
59	DLG5 Isoform 1 of Disks large homolog 5
60	RAPGEF1 guanine nucleotide-releasing factor 2 isoform b
61	PGRMC2 cDNA FLJ34422
62	C1orf162 Isoform 1 of Transmembrane protein C1orf162

* Proteins that are also pulled downed with control IgG have been excluded

bioRxiv preprint doi: https://doi.org/10.1101/2025.10.06.680735; this version posted October 6, 2025. The copyright holder for this preprint (which was not certified by peer review) is the author/funder, who has granted bioRxiv a license to display the preprint in perpetuity. It is made available under aCC-BY 4.0 International license.

Supplementary Table 2. Oligo-based DNA nick or flap substrates representing different intermediates during OFM

Substrate diagram	Structure features of the substrate
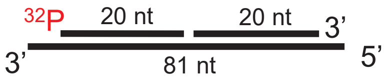	The DNA nick substrate
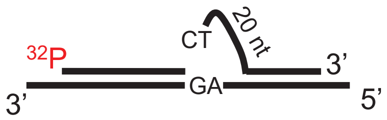	The DNA flap substrate with the ssDNA flap strand possibly annealing to the template strand via a 2 nt microhomology sequences.
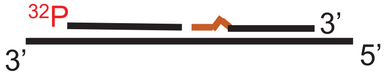	The DNA nick substrate with DNA mismatch 2 nt downstream the nick.

bioRxiv preprint doi: https://doi.org/10.1101/2025.10.06.680735; this version posted October 6, 2025. The copyright holder for this preprint (which was not certified by peer review) is the author/funder, who has granted bioRxiv a license to display the preprint in perpetuity. It is made available under aCC-BY 4.0 International license.

Supplementary Table S3: List of oligos for substrate ligation assay

Name	Sequence
Template	5’GTTAAGATAGGTCTGCTTGGGATGTCAAGCAGTCCTAACTGGAAAT CTAG-CTCTGTGGAGTTGAGGCAGAGTCCTTAAGC-3’
Upstream	5’- TCTGCCTCAACTCCACAGAG -3’
Downstream	5’PO4- CTAGATTTCCAGTTAGGACT -3’
Downstream mismatch	5’PO4- CTCCATTTCCAGTTAGGACT -3’
Down-stream flap	5’PO4- CTCGTAAGCTATAGGGGAAAAAAGATTTCCAGTTAGGACT -3’

bioRxiv preprint doi: https://doi.org/10.1101/2025.10.06.680735; this version posted October 6, 2025. The copyright holder for this preprint (which was not certified by peer review) is the author/funder, who has granted bioRxiv a license to display the preprint in perpetuity. It is made available under aCC-BY 4.0 International license.

Table S4. The SNVs from R192Q mapping to COSMIC signatures

Signature	Similarity	Annotation
SBS5	0.797	clock-like signature
SBS40	0.783	Unknown
SBS89	0.752	Unknown
SBS3	0.734	Tobacco smoking
SBS25	0.644	Chemotherapy treatment
SBS8	0.563	Unknown
SBS44	0.538	Defective DNA mismatch repair
SBS18	0.534	Damage by reactive oxygen species
SBS4	0.528	Unknown (clock-like signature)
SBS32	0.51	Azathioprine treatment
SBS9	0.502	Polimerase eta somatic hypermutation activity
SBS29	0.493	Tobacco chewing
SBS6	0.491	Defective DNA mismatch repair
SBS39	0.481	Unknown
SBS37	0.479	Unknown
SBS24	0.471	Aflatoxin exposure
SBS42	0.469	Haloalkane exposure
SBS87	0.447	Thiopurine chemotherapy treatment
SBS30	0.444	Defective DNA base excision repair due to NTHL1 mutations
SBS12	0.44	Unknown
SBS26	0.439	Defective DNA mismatch repair
SBS36	0.434	Defective DNA base excision repair due to MUTYH mutations
SBS31	0.419	Platinum chemotherapy treatment
SBS35	0.407	Platinum chemotherapy treatment
SBS15	0.395	Defective DNA mismatch repair
SBS19	0.395	Unknown
SBS84	0.392	Activity of activation-induced cytidine deaminase (AID)
SBS41	0.38	Unknown
SBS20	0.368	Concurrent POLD1 mutations and defective DNA mismatch repair
SBS11	0.363	Temozolomide treatment
SBS23	0.363	Unknown
SBS1	0.359	Activity of APOBEC family of cytidine deaminases
SBS16	0.349	Unknown
SBS7b	0.346	Ultraviolet light exposure
SBS85	0.341	Indirect effects of activation-induced cytidine deaminase (AID)
SBS33	0.317	Unknown
SBS88	0.315	Colibactin exposure (E.coli bacteria carrying pks pathogenicity island)
SBS14	0.311	Concurrent polymerase epsilon mutation and defective DNA mismatch repair
SBS7a	0.271	Ultraviolet light exposure
SBS21	0.261	Defective DNA mismatch repair
SBS10b	0.25	Polymerase epsilon exonuclease domain mutations
SBS86	0.25	Unknown chemotherapy treatment
SBS7d	0.248	Ultraviolet light exposure
SBS2	0.218	Defective homologous recombination DNA damage repair
SBS38	0.21	Indirect effect of ultraviolet light
SBS22	0.206	Aristolochic acid exposure
SBS7c	0.205	Ultraviolet light exposure
SBS10a	0.199	Polymerase epsilon exonuclease domain mutations
SBS28	0.155	Unknown
SBS17a	0.148	Unknown
SBS13	0.128	Activity of APOBEC family of cytidine deaminases
SBS34	0.109	Unknown
SBS17b	0.106	Unknown
SBS90	0.09	Duocarmycin exposure

bioRxiv preprint doi: https://doi.org/10.1101/2025.10.06.680735; this version posted October 6, 2025. The copyright holder for this preprint (which was not certified by peer review) is the author/funder, who has granted bioRxiv a license to display the preprint in perpetuity. It is made available under aCC-BY 4.0 International license.

Table S5. The SNVs from *jmjd1b*$^{-/-}$ mapping to COSMIC signatures

Signature	Similarity	Annotation
SBS5	0.805	clock-like signature
SBS26	0.646	Defective DNA mismatch repair
SBS6	0.63	Defective DNA mismatch repair
SBS87	0.61	Thiopurine chemotherapy treatment
SBS3	0.586	Tobacco smoking
SBS40	0.58	Unknown
SBS25	0.578	Chemotherapy treatment
SBS12	0.575	Unknown
SBS44	0.556	Defective DNA mismatch repair
SBS1	0.552	Activity of APOBEC family of cytidine deaminases
SBS37	0.499	Unknown
SBS32	0.488	Azathioprine treatment
SBS89	0.484	Unknown
SBS15	0.473	Defective DNA mismatch repair
SBS30	0.451	Defective DNA base excision repair due to NTHL1 mutations
SBS31	0.435	Platinum chemotherapy treatment
SBS42	0.421	Haloalkane exposure
SBS9	0.421	Polimerase eta somatic hypermutation activity
SBS33	0.414	Unknown
SBS23	0.413	Unknown
SBS19	0.409	Unknown
SBS7b	0.397	Ultraviolet light exposure
SBS11	0.394	Temozolomide treatment
SBS39	0.394	Unknown
SBS21	0.393	Defective DNA mismatch repair
SBS84	0.384	Activity of activation-induced cytidine deaminase (AID)
SBS16	0.344	Unknown
SBS20	0.339	Concurrent POLD1 mutations and defective DNA mismatch repair
SBS24	0.323	Aflatoxin exposure
SBS41	0.307	Unknown
SBS7d	0.304	Ultraviolet light exposure
SBS7a	0.302	Ultraviolet light exposure
SBS35	0.301	Platinum chemotherapy treatment
SBS4	0.3	Unknown (clock-like signature)
SBS18	0.298	Damage by reactive oxygen species
SBS29	0.298	Tobacco chewing
SBS8	0.297	Unknown
SBS17a	0.283	Unknown
SBS85	0.282	Indirect effects of activation-induced cytidine deaminase (AID)
SBS10b	0.262	Polymerase epsilon exonuclease domain mutations
SBS88	0.25	Colibactin exposure (E.coli bacteria carrying pks pathogenicity island)
SBS86	0.218	Unknown chemotherapy treatment
SBS14	0.217	Concurrent polymerase epsilon mutation and defective DNA mismatch repair
SBS36	0.195	Defective DNA base excision repair due to MUTYH mutations
SBS2	0.191	Defective homologous recombination DNA damage repair
SBS7c	0.165	Ultraviolet light exposure
SBS38	0.146	Indirect effect of ultraviolet light
SBS22	0.127	Aristolochic acid exposure
SBS10a	0.072	Polymerase epsilon exonuclease domain mutations
SBS13	0.057	Activity of APOBEC family of cytidine deaminases
SBS34	0.053	Unknown
SBS28	0.05	Unknown
SBS17b	0.049	Unknown
SBS90	0.039	Duocarmycin exposure

bioRxiv preprint doi: https://doi.org/10.1101/2025.10.06.680735; this version posted October 6, 2025. The copyright holder for this preprint (which was not certified by peer review) is the author/funder, who has granted bioRxiv a license to display the preprint in perpetuity. It is made available under aCC-BY 4.0 International license.

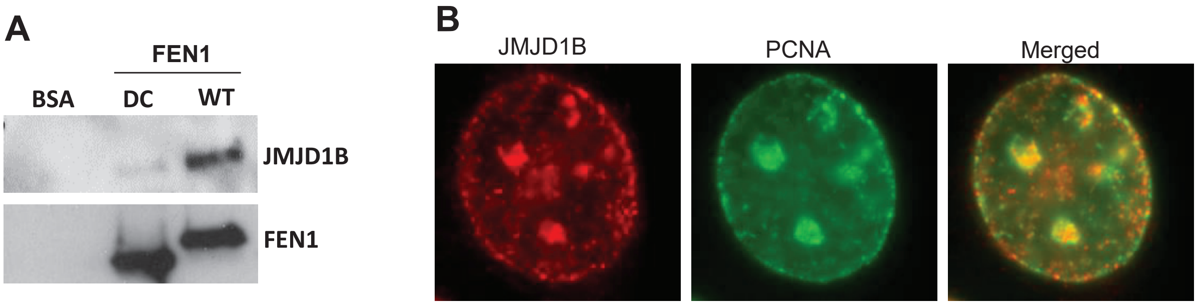

Supplementary Figure S1. JMJD1B interacts with FEN1 and is associated with replication forks in HeLa cells. (A) co-pulled JMJD1B with purified WT or C-terminal deletion (DC) FEN1 proteins. **(B)** JMJD1B foci co-localizes with PCNA foci in HeLa cells.

bioRxiv preprint doi: https://doi.org/10.1101/2025.10.06.680735; this version posted October 6, 2025. The copyright holder for this preprint (which was not certified by peer review) is the author/funder, who has granted bioRxiv a license to display the preprint in perpetuity. It is made available under aCC-BY 4.0 International license.

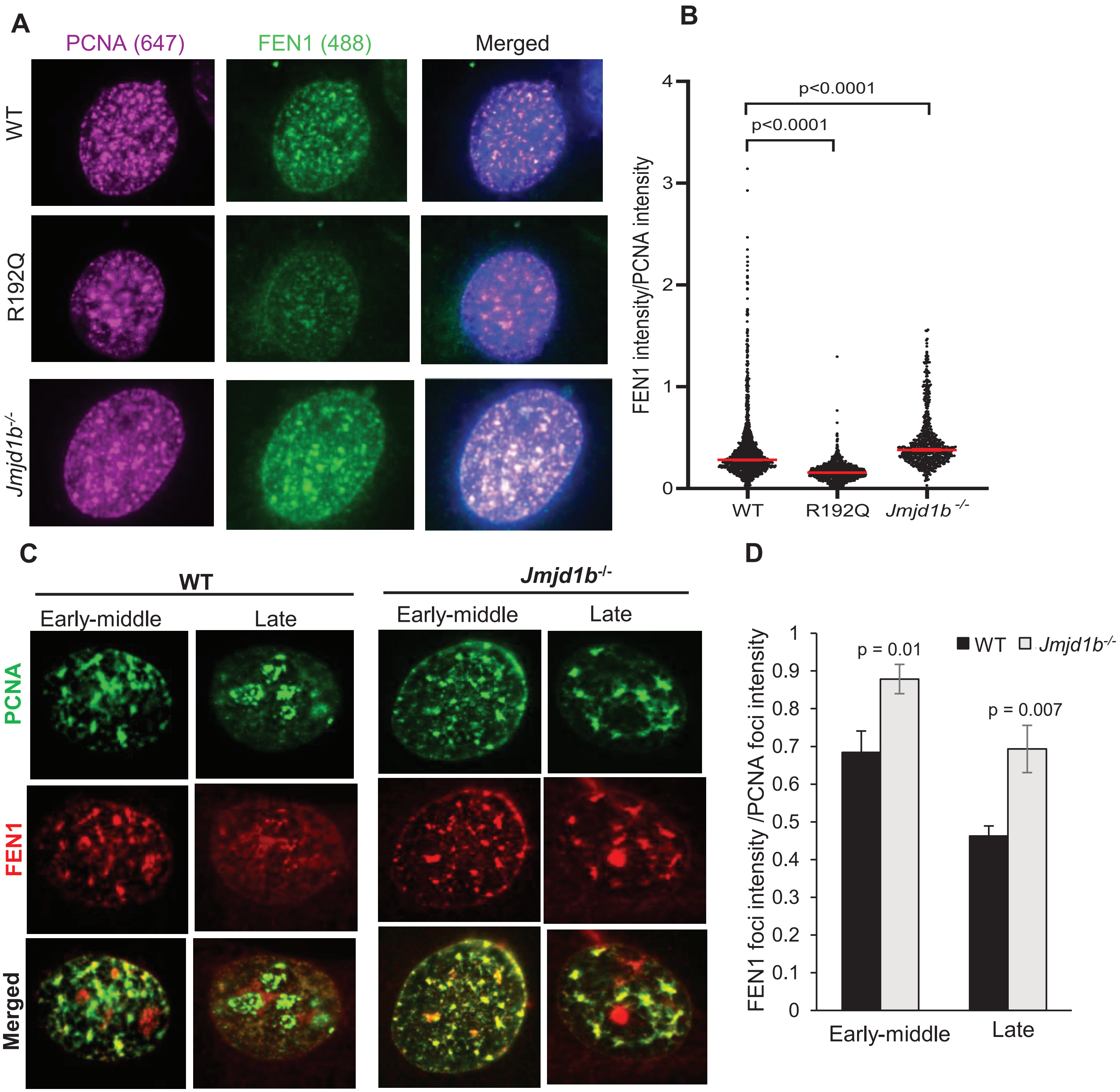

Supplementary Figure S2. Co-IF staining shows FEN1 and PCNA co-localization in WT, R192Q, or *Jmjd1b*$^{-/-}$ MEFs. (A) FEN1 co-localization with PCNA in WT, R192Q and *Jmjd1b*$^{-/-}$ MEFs. **(B)** FEN1/PCNA foci intensity ratio in WT, R192Q and *Jmjd1b*$^{-/-}$ MEFs. Mean ± SEM are indicated. P value from Student t-test. **(C)** FEN1 co-localization with PCNA at various stages of S phase in WT and Jmjd1b-/- MEFs. **(D)** FEN1/PCNA foci intensity ratio in indicated stages of S phase in WT and Jmjd1b-/- MEFs. Mean ± SEM are indicated. P value from Student t-test.

bioRxiv preprint doi: https://doi.org/10.1101/2025.10.06.680735; this version posted October 6, 2025. The copyright holder for this preprint (which was not certified by peer review) is the author/funder, who has granted bioRxiv a license to display the preprint in perpetuity. It is made available under aCC-BY 4.0 International license.

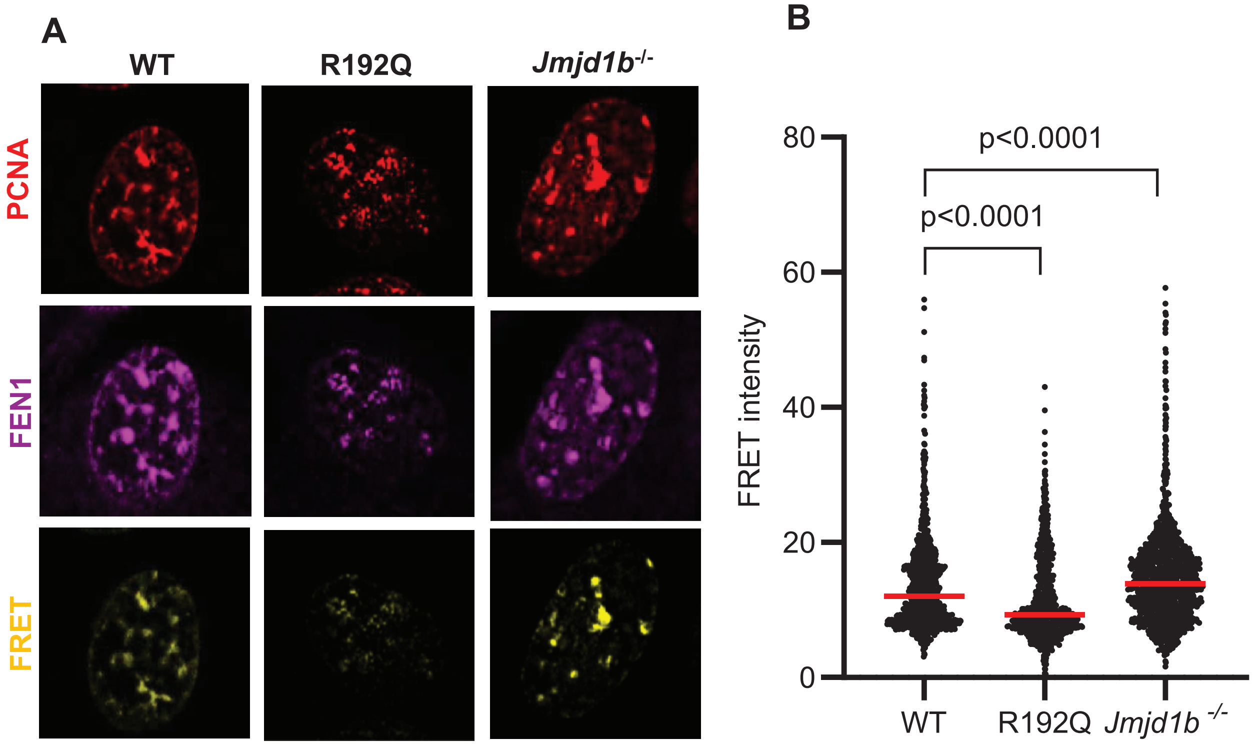

Supplementary Figure S3. FEN1-PCNA FRET the overall level of the FEN1-PCNA complex in WT, R192Q, or *Jmjd1b*$^{-/-}$ MEFs. (A) representative images for the Alexa 568 (PCNA), Alexa 647 (FEN1), or the FRET channel. **(B)** The FRET intensity, which was corrected with the signals from background and bleed-through, in WT, R192Q and *Jmjd1b*$^{-/-}$ MEFs. Mean ± SEM are indicated. P value from Student t-test.

bioRxiv preprint doi: https://doi.org/10.1101/2025.10.06.680735; this version posted October 6, 2025. The copyright holder for this preprint (which was not certified by peer review) is the author/funder, who has granted bioRxiv a license to display the preprint in perpetuity. It is made available under aCC-BY 4.0 International license.

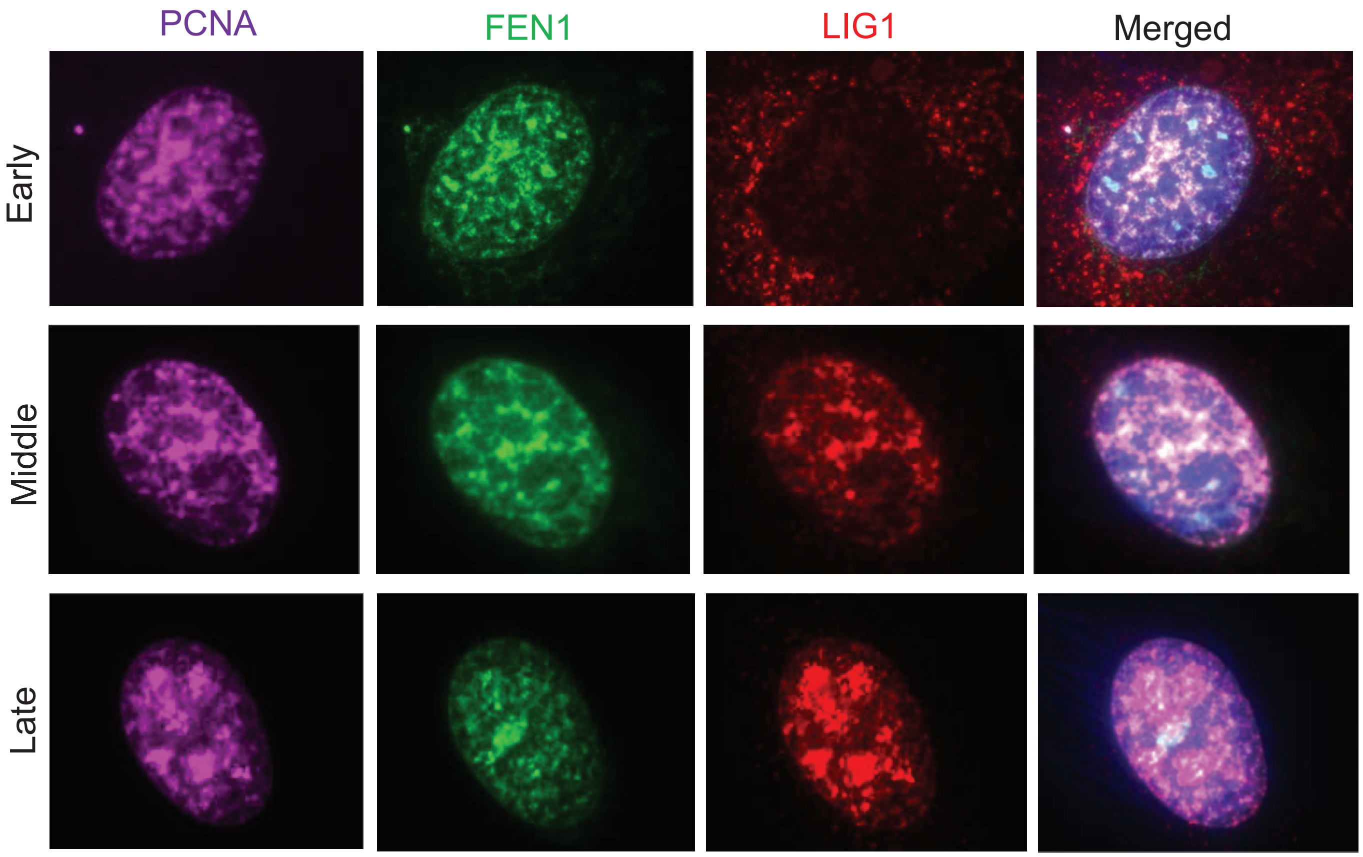

Supplementary Figure S4. FEN1-PCNA-LIG1 co-localization in WT MEFs. Representative images for the Alexa 647 (PCNA), Alexa 488 (FEN1) and Alexa 568 (LIG1), and the merged view.

bioRxiv preprint doi: https://doi.org/10.1101/2025.10.06.680735; this version posted October 6, 2025. The copyright holder for this preprint (which was not certified by peer review) is the author/funder, who has granted bioRxiv a license to display the preprint in perpetuity. It is made available under aCC-BY 4.0 International license.

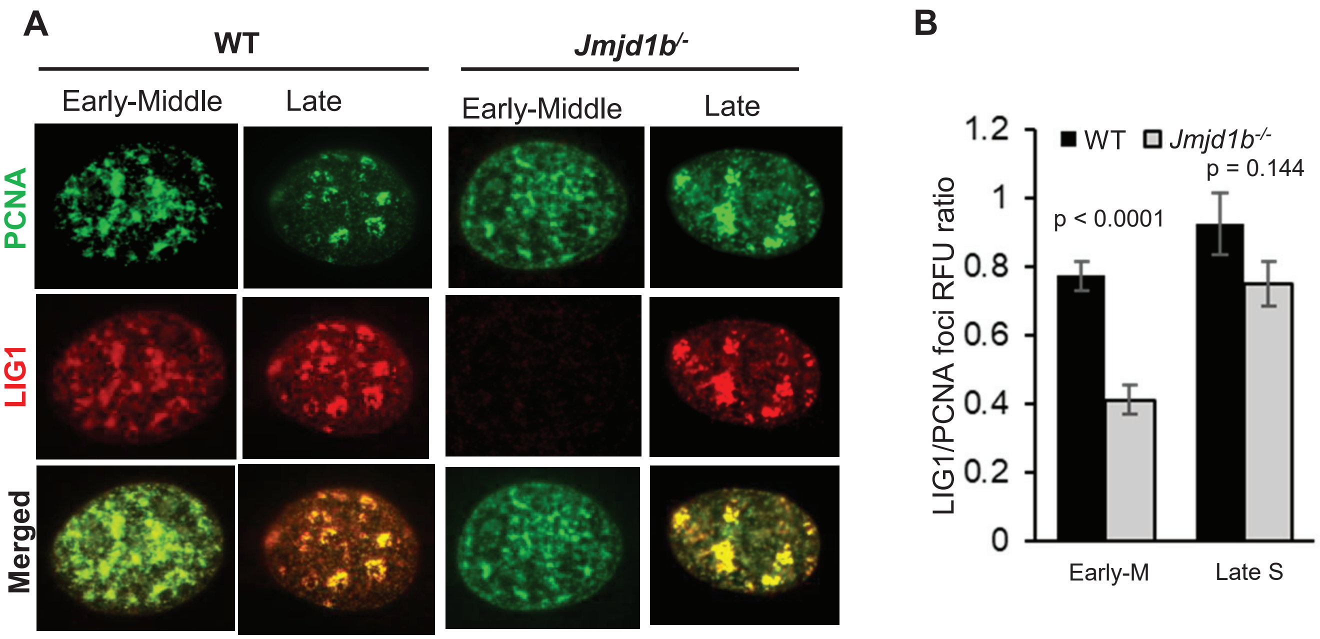

Supplementary Figure S5. PCNA LIG1 co-localization in WT and *Jmjd1b*$^{-/-}$ cells. (A) LIG1 co-localization with PCNA at various stages of S phase in WT and *Jmjd1b*$^{-/-}$ MEFs. **(B)** LIG1/PCNA foci intensity ratio in indicated stages of S phase in WT and *Jmjd1b*$^{-/-}$ MEFs. Mean ± SEM are indicated. P value from Student t-test.

bioRxiv preprint doi: https://doi.org/10.1101/2025.10.06.680735; this version posted October 6, 2025. The copyright holder for this preprint (which was not certified by peer review) is the author/funder, who has granted bioRxiv a license to display the preprint in perpetuity. It is made available under aCC-BY 4.0 International license.

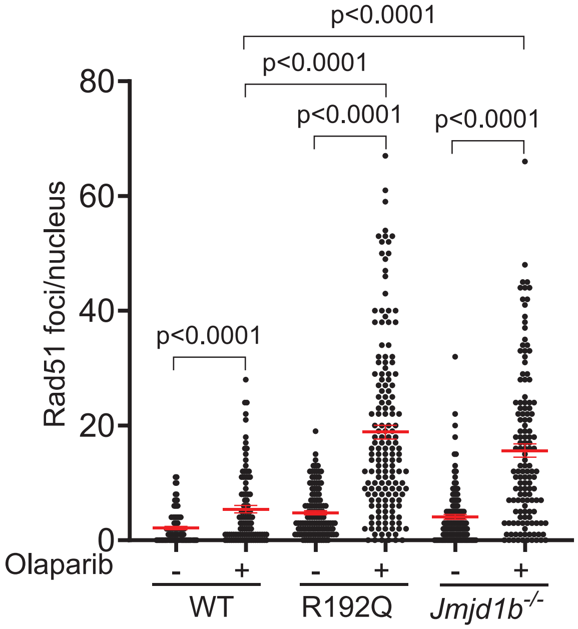

Supplementary Figure S6. RAD51 foci in WT, R192Q, and *Jmjd1b*$^{-/-}$ MEFs without or with Olaprib treatment. Quantification of RAD51 foci in WT, FEN1 R192Q, and *Jmjd1b*$^{-/-}$ MEFs (± Olaparib, 10 μM, 16 h) using the Image J program. Mean ± SEM. are indicated. P values are calculated using the student's t-test.

bioRxiv preprint doi: https://doi.org/10.1101/2025.10.06.680735; this version posted October 6, 2025. The copyright holder for this preprint (which was not certified by peer review) is the author/funder, who has granted bioRxiv a license to display the preprint in perpetuity. It is made available under aCC-BY 4.0 International license.

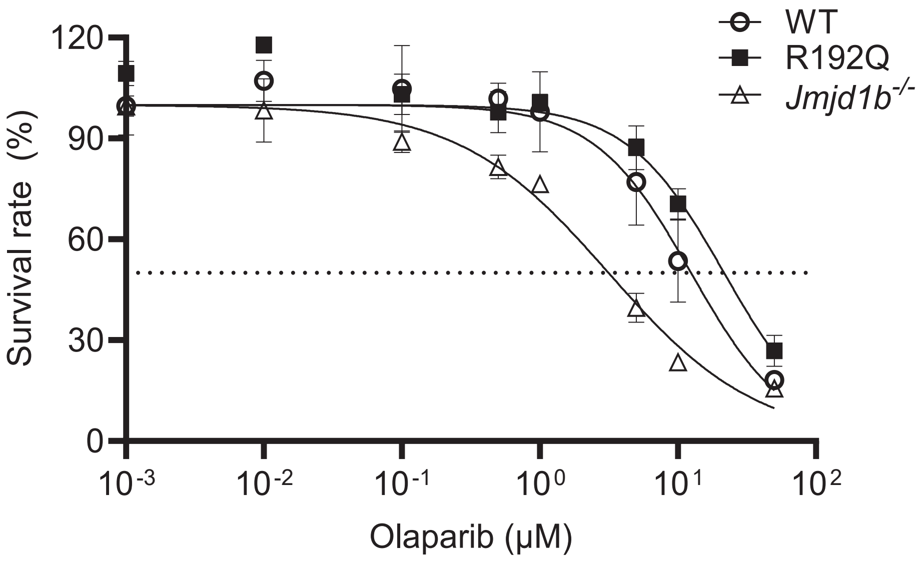

Supplementary Figure S7. Survival curves of WT, R192Q, *Jmjd1b*$^{-/-}$, and FEN1 S187A cells to Olaprib. Cells were seeded in 12-well plates and treated with varying concentrations of Olaparib for 96 h. Viable cells were counted and survival rate was calculated by dividing the number of surviving cells in a treatment group by those in the untreated control. Values are means ± SEM of four independent assays.

bioRxiv preprint doi: https://doi.org/10.1101/2025.10.06.680735; this version posted October 6, 2025. The copyright holder for this preprint (which was not certified by peer review) is the author/funder, who has granted bioRxiv a license to display the preprint in perpetuity. It is made available under aCC-BY 4.0 International license.

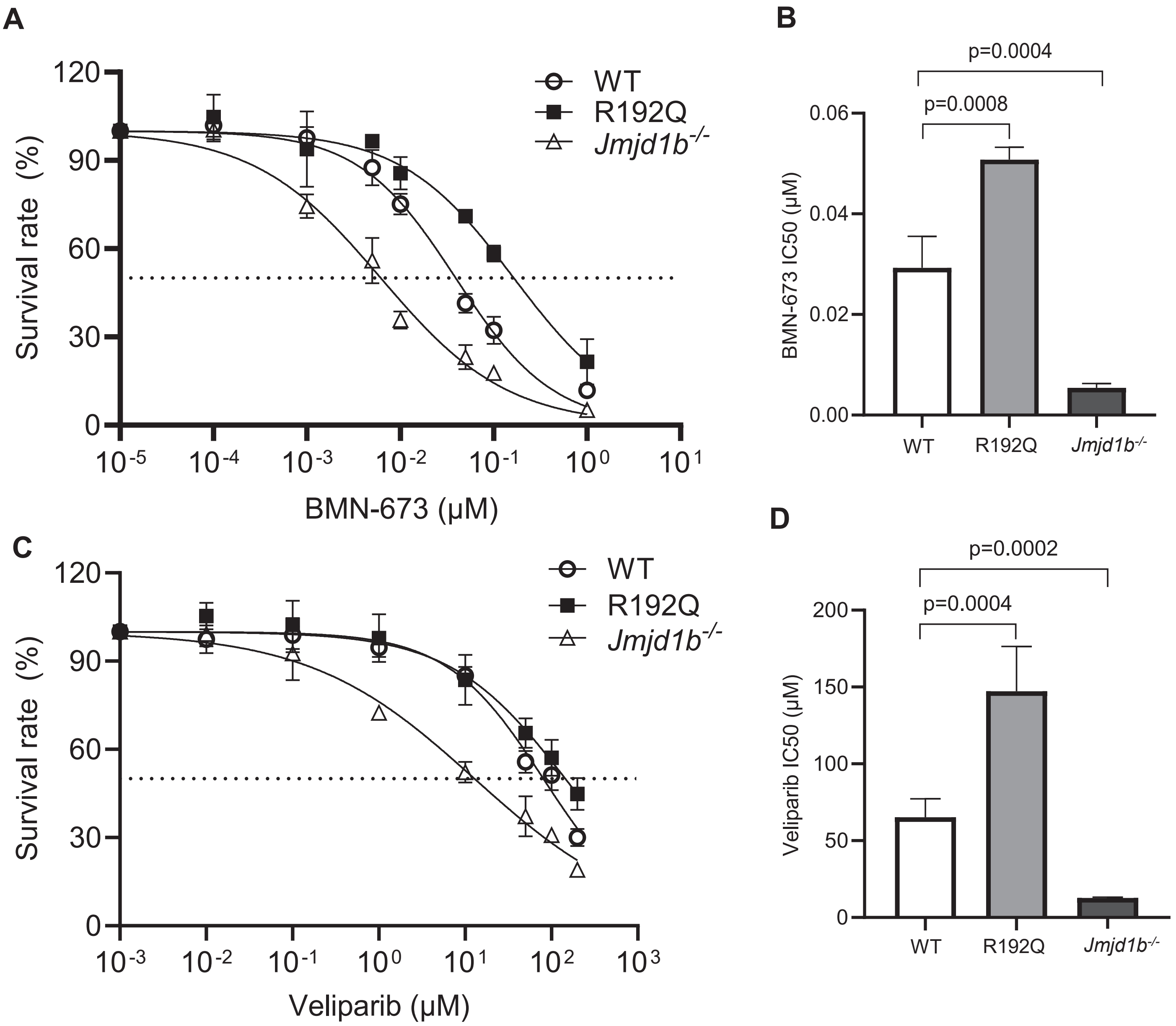

Supplementary Figure S8. Sensitivity of WT, R192Q, *Jmjd1b*$^{-/-}$, and FEN1 S187A cells to PARP inhibitors BMN673 or veliparib. Cells were seeded in 12-well plates and treated with varying concentrations of BMN673 (A, B), or veliparib (C,D) for 96 h. Viable cells were counted and survival rate was calculated by dividing the number of surviving cells in a treatment group by those in the untreated control. Panel A or C are survival curves of different cells treated with BMN673 or veliparib respectively. Panel B and D are the calculated IC50 based on the survival curve. Values are means ± SEM of four independent assays. P value from Student t-test.

bioRxiv preprint doi: https://doi.org/10.1101/2025.10.06.680735; this version posted October 6, 2025. The copyright holder for this preprint (which was not certified by peer review) is the author/funder, who has granted bioRxiv a license to display the preprint in perpetuity. It is made available under aCC-BY 4.0 International license.

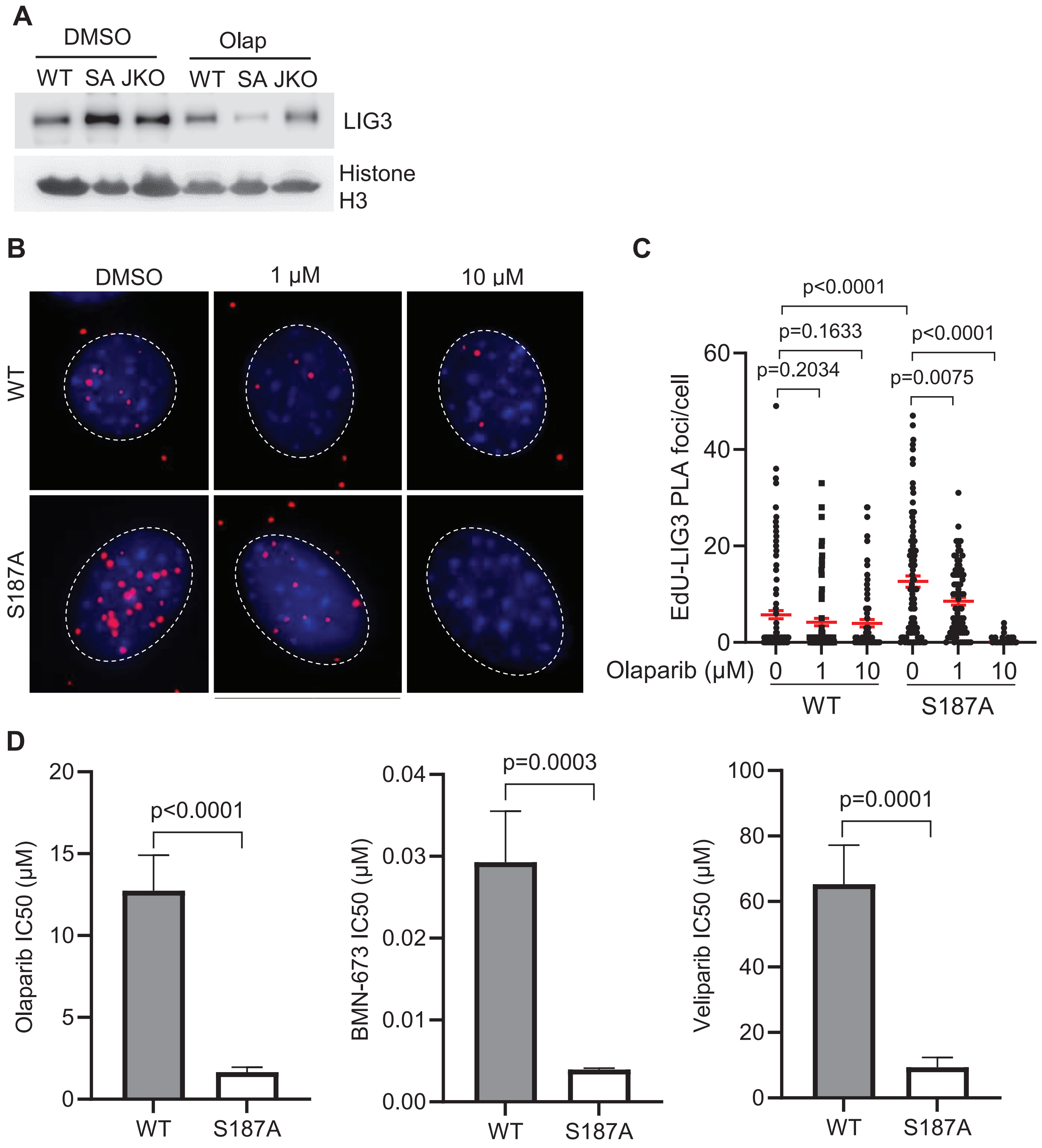

Supplementary Figure S9. Chromatin- and replication fork-associated LIG3 in MEFs without or with Olaparib-treatment. (A) Western blot analysis of chromatin-associated LIG3 from WT, S187A (SA), or *Jmjd1b*$^{-/-}$ (JKO) cells treated with DMSO or Olaparib (10 μM, 16 h). Histone H3 was used as a loading control. **(B)** Representative images and **(C)** Quantification of Edu-LIG3 PLA foci in WT or S187A cells treated with DMSO or Olaparib (1, 10 μM, 16 h). Nuclei were stained with DAPI (Blue). Mean ± SEM are indicated. P value from Student t-test. **(D)** Sensitivity to cell killing of WT or S187A cells by Olaparib, BMN-673, or Veliparib. Values are mean ± SEM of four independent assays. P value from Student t-test.

bioRxiv preprint doi: https://doi.org/10.1101/2025.10.06.680735; this version posted October 6, 2025. The copyright holder for this preprint (which was not certified by peer review) is the author/funder, who has granted bioRxiv a license to display the preprint in perpetuity. It is made available under aCC-BY 4.0 International license.

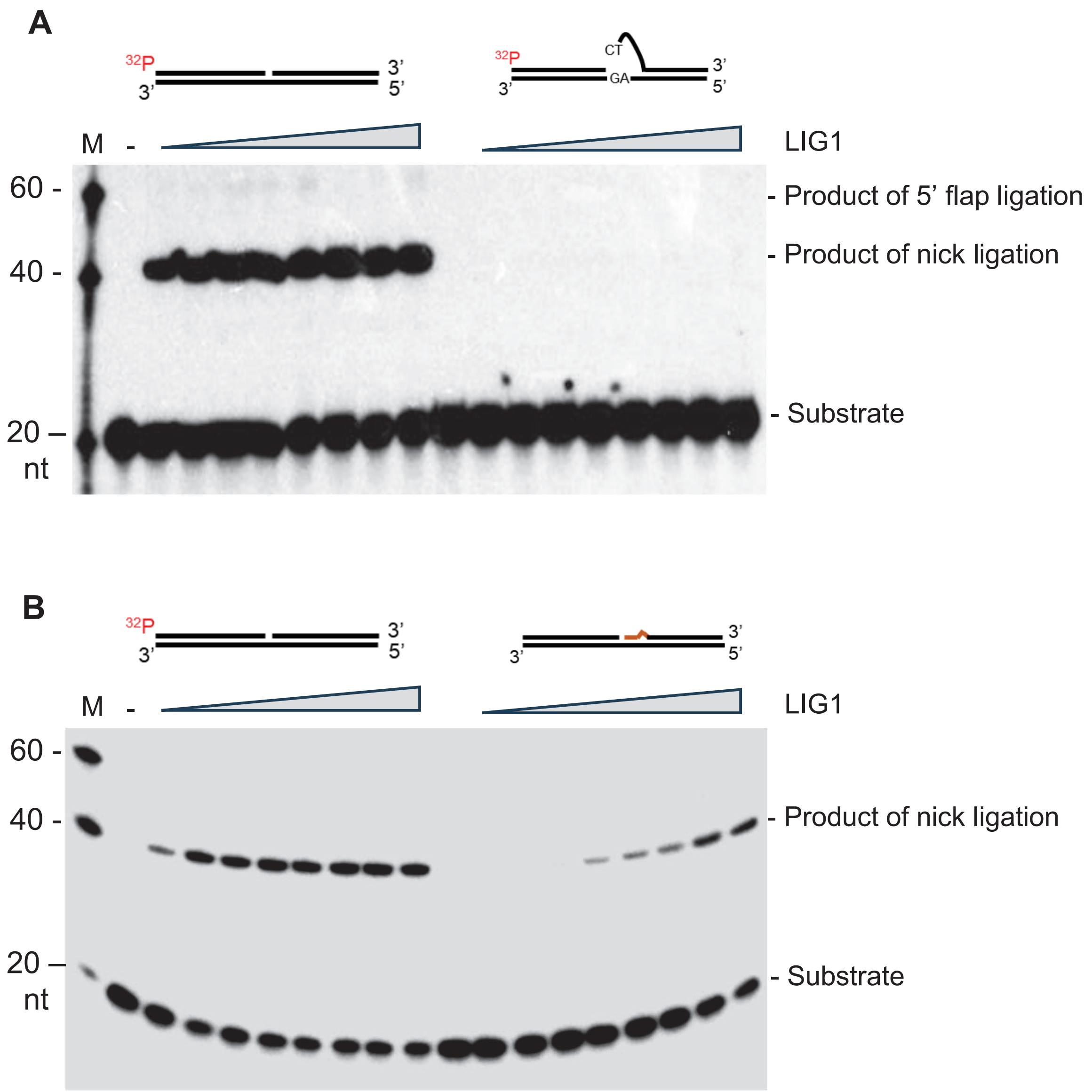

Supplementary Figure S10. DNA ligation of DNA nick and flap substrates by LIG1. Representative denaturing PAGE image shows ligation of (A) 5' flap substrates and (B) nick substrates by purified recombinant LIG1 of varying concentrations (5, 10, 20, 30, 40, 50, 100, 150 nM).

bioRxiv preprint doi: https://doi.org/10.1101/2025.10.06.680735; this version posted October 6, 2025. The copyright holder for this preprint (which was not certified by peer review) is the author/funder, who has granted bioRxiv a license to display the preprint in perpetuity. It is made available under aCC-BY 4.0 International license.

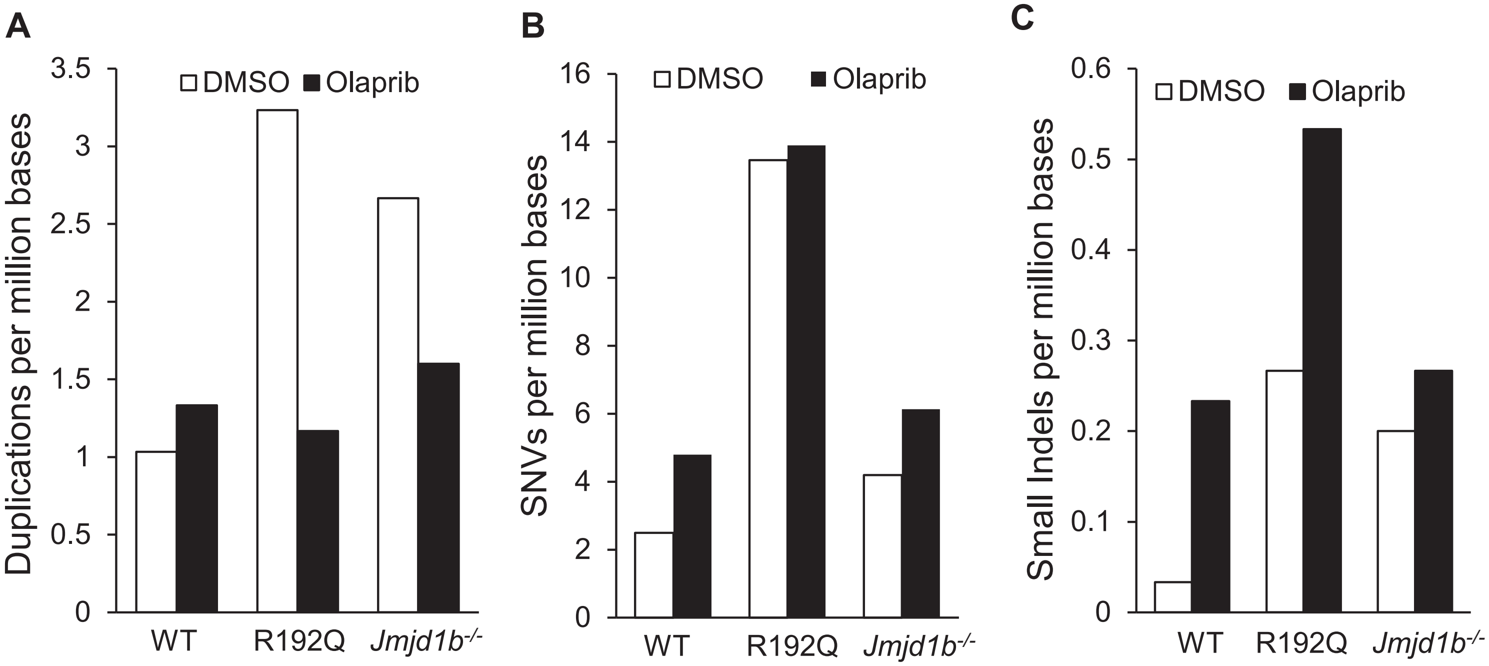

Figure S11. Frequency of somatic duplications, single nucleotide variation (SNV), small insertion/deletion (IN/DEL) in WT, R192Q, *Jmjd1b*$^{-/-}$ cells with or without Olaparib treatment. Cells were treated with DMSO (control) or Olaprib for 72 hours. WES was carried out and somatic mutation frequence for (A) duplications, (B) SNVs, or (C) small Indels were calculated.

bioRxiv preprint doi: https://doi.org/10.1101/2025.10.06.680735; this version posted October 6, 2025. The copyright holder for this preprint (which was not certified by peer review) is the author/funder, who has granted bioRxiv a license to display the preprint in perpetuity. It is made available under aCC-BY 4.0 International license.

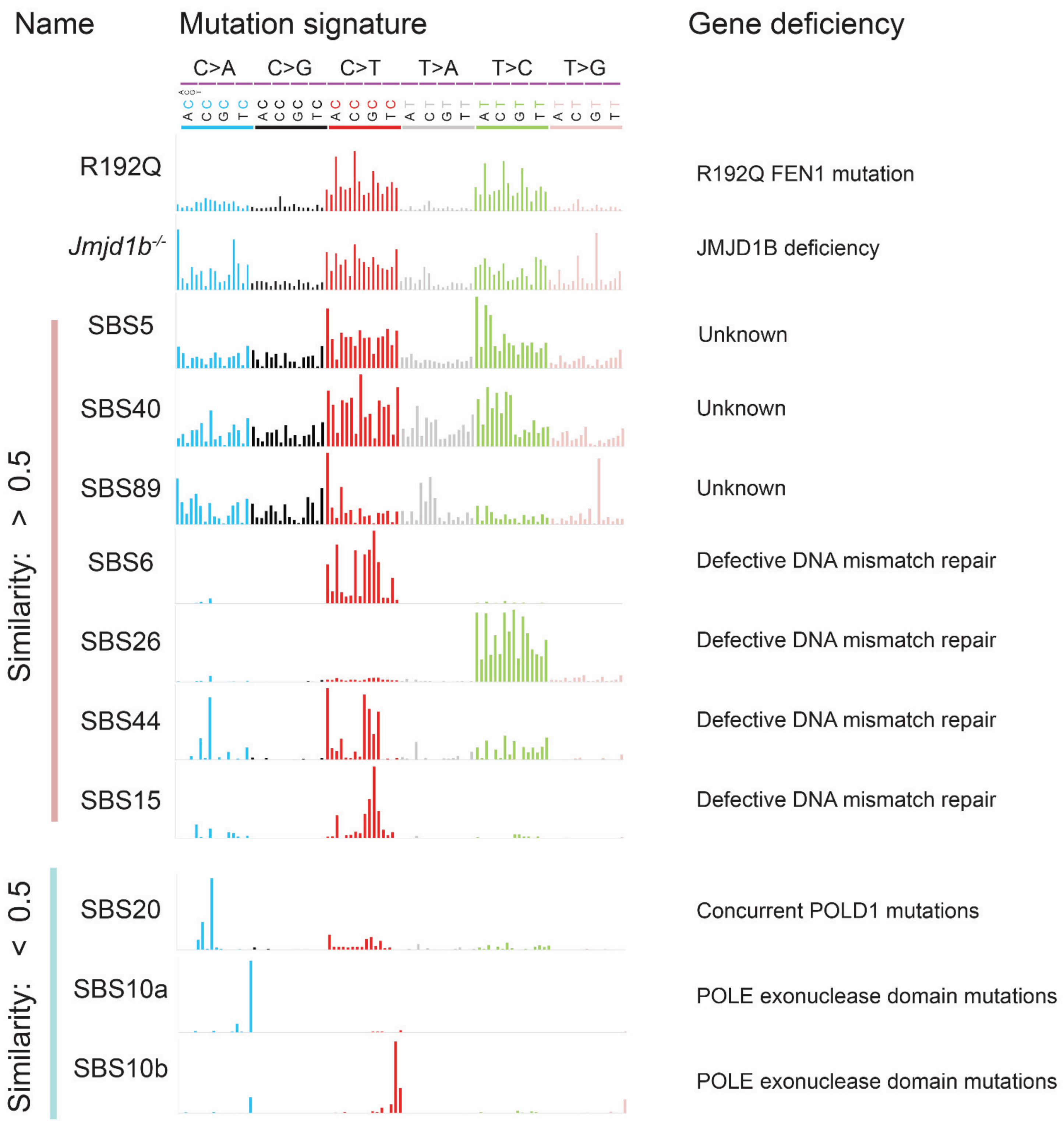

Figure S12. Mutation signatures in human cancers with high and low similarity to those in R192Q and *Jmjd1b*$^{-/-}$ MEFs.
